# The Role of Galectin-9 as Mediator of Atopic Dermatitis: Effect on Keratinocytes

**DOI:** 10.3390/cells10040947

**Published:** 2021-04-20

**Authors:** Mab P. Corrêa, Libnah L. Areias, Rebeca D. Correia-Silva, Solange C. G. P. D’Ávila, Andréia M. Leopoldino, Karin V. Greco, Cristiane D. Gil

**Affiliations:** 1 Programa de Pós-Graduação em Biociências, Instituto de Biociências Letras e Ciências Exatas, Universidade Estadual Paulista (UNESP), São José do Rio Preto, SP 15054-000, Brazil; mabiiic@gmail.com; 2 Departamento de Morfologia e Genética, Escola Paulista de Medicina, Universidade Federal de São Paulo (UNIFESP), São Paulo, SP 04023-900, Brazil; libnahleal@gmail.com (L.L.A.); beca97c@gmail.com (R.D.C.-S.); 3 Departamento de Patologia e Medicina Forense, Faculdade de Medicina de São José do Rio Preto (FAMERP), São José do Rio Preto, SP 15090-000, Brazil; spdpatho@famerp.br; 4 Departamento de Análises Clínicas, Toxicológicas e Bromatológicas, Faculdade de Ciências Farmacêuticas de Ribeirão Preto, Universidade de São Paulo (USP), Ribeirão Preto, SP 14040-903, Brazil; andreiaml@usp.br; 5 Division of Surgery and Interventional Science, The Griffin Institute, University College London (UCL), London HA13UJ, UK; k.greco@ucl.ac.uk

**Keywords:** skin inflammation, atopic dermatitis, galectin, mast cell, eosinophil, keratinocyte, IL-6

## Abstract

Galectin-9 (Gal-9) is a beta-galactoside-binding protein with a variety of biological functions related to immune response. However, in allergic diseases, its mechanism of action is not fully understood. This study evaluates the expression pattern of Gal-9 in patients with atopic dermatitis (AD), in ovalbumin (OVA)-induced experimental atopic dermatitis (AD) in mice, as well as its effect on human keratinocytes. The skin of OVA-immunized BALB/c mice was challenged with drops containing OVA on days 11, 14–18, and 21–24. HaCaT cells were cultured in the following experimental conditions: control (growth medium only) or stimulated with TNF-α/IFN-γ, or IL-4, or IL-17 with or without Gal-9 treatment. AD was characterized by increased levels of Gal-9 in mouse and human skin, especially in the epidermis, and with a marked influx of Gal-9 positive eosinophils and mast cells compared to the control group. Gal-9 showed an immunomodulatory effect on keratinocytes by decreasing the release of IL-6 by IL-4-stimulated keratinocytes or increasing the IL-6 and RANTES levels by IL-17- or TNF-α/IFN-γ-stimulated cells, respectively. Under IL-17, Gal-9 treatment also altered the proliferation rate of cells. Overall, increased levels of Gal-9 in AD skin contribute to the control of inflammatory response and the proliferative process of keratinocytes, suggesting this lectin as a relevant therapeutic target.

## 1. Introduction

Atopic dermatitis (AD), also known as atopic eczema, represents the most common inflammation of the skin, characterized by reddish lesions that itch, peel, and sometimes get wet. AD has an early onset and can affect 25% of children, with about 10% carrying the disease into adulthood [1,2]. AD patients have a higher incidence of bacterial, fungal, or viral infections due to epithelial barrier dysfunction in skin lesions. AD etiology is not completely clear, and it seems to be multifactorial as there is evidence that genetic predisposition and a family history of atopies can influence the onset of this disease. The barrier dysfunction occurs due to an impairment in keratinocytes’ terminal differentiation, which permits the penetration of antigens. Furthermore, chronic pruritus and change in the pattern of resident microbiota support colonization by *Staphylococcus aureus* in the lesions [1,3].

Complex immune responses that involve chronic skin inflammations show the challenge faced by doctors in the clinical diagnosis of these pathologies and the consequent application of appropriate treatment. The study of biomarkers, therefore, is paramount to better define the heterogeneity of these pathologies and contribute to the development of bespoke therapies. In this study, we focused on the galectin-9 (Gal-9), also known as ecalectin, tumor antigen HOM-HD-21, and urate transporter/channel protein. Gal-9 is a 40-kDa protein capable of regulating the inflammatory response belonging to a family of proteins that recognize β-galactosides in several cell surface receptors and extracellular matrix (ECM) receptors [4]. In mammals, 15 members are described and classified in three subfamilies: (a) the prototypical galectins (1, 2, 5, 7, 10, 11, 13, 14, and 15), having a single type of carbohydrate recognition domain (CRD) displayed either as monomers or dimerizing to form homodimers with two CRDs; (b) tandem-repeats with repetitions in sequence (galectins 4, 6, 8, 9, and 12) that share two distinct and homologous CRDs; (c) the chimeric type, with Gal-3 as single member, formed of a collagenlike N-terminal domain and a C-terminal domain containing a single CRD [4,5].

It is shown that patients with AD have high levels of Gal-9 both in the serum and epidermis, with even higher doses in severe cases, which tends to decrease after treatment, suggesting proinflammatory action [6]. Contrarily, in mouse experimental models, exogenous Gal-1 and Gal-9 play anti-inflammatory roles in both AD and allergic contact dermatitis (a Th1/Th17-profile disease), counteracting the inflammation and decreasing the production of IFN-γ and IL-17, but not of Th2 cytokines [7,8].

In relation to the mechanisms of action of the allergy, Gal-9 seems to play a dual role, by either sometimes regulating or activating the cellular inflammatory response. Studies have shown that Gal-9 is able to bind to IgE and prevent the formation of the antigen–antibody complex and mast cell degranulation with the consequent release of proinflammatory mediators [9]. On the other hand, others have shown that incubation of human mast cells (HMC-1 strain) with Gal-9 was able to induce release of IL-6, IL-8, and MCP-1 via the activation of the ERK1/2 cascade [10].

In an in vitro chemotaxis assay, Gal-9 can act as a chemo-attractive factor for human eosinophils [11]. In human skin, IFN-γ provokes an enhanced expression of Gal-9 by the fibroblasts while the opposite effect is observed on keratinocytes [12]. The results suggest that a fibroblast-induced Gal-9 expression in dermis would favor eosinophil chemotaxis in an IFN-γ-modulated type of inflammation.

Based on these paradoxical roles of Gal-9 in allergic reactions, especially in the skin, this study evaluates the expression of this lectin both in vivo, using a murine AD model and in vitro, showing its role on keratinocytes under different inflammatory stimuli. We also assessed the expression of Gal-9 in biopsies from patients with a confirmed clinical diagnosis of AD.

## 2. Materials and Methods

### 2.1. Experimental Model of Atopic Dermatitis

**Figure 1:**
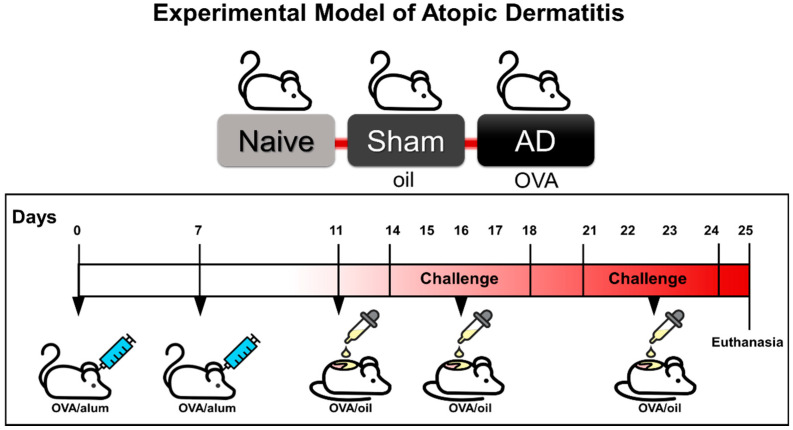
Experimental model of atopic dermatitis (AD). On days 0 and 7, male BALB/c mice were immunized with a subcutaneous injection of 5 µg of ovalbumin (OVA) and 10 mg/mL of the aluminum hydroxide adjuvant (alum). On days 11, 14 to 18, and 21 to 24, dorsal shaved flanks of animals were challenged with 250 µg of OVA diluted in 50 µL of Johnson’s Baby^®^ oil. Sham animals received only sterile saline (days 0 and 7) and oil (days 11, 14–18, 21–24), while the naive group was only manipulated. After 24 h of the last OVA challenge, animals were euthanized for skin analysis.

The mouse skin samples were obtained following our previous investigations [13] and approved by the Ethics Committee in Animal Experimentation of the Federal University of São Paulo—UNIFESP (CEP 1906060115/2015). In summary, on days 0 and 7, male BALB/c mice were immunized with a subcutaneous injection of 5 µg of ovalbumin (OVA; V grade; Sigma-Aldrich, St Louis, MO, USA) and 10 mg/mL of the aluminum hydroxide adjuvant (ALUM; Sigma-Aldrich) diluted in 200 µL of sterile saline (Figure 1). On days 11, 14 to 18, and 21 to 24, dorsal shaved flanks of animals were challenged with 250 µg of OVA diluted in 50 µL of Johnson’s Baby^®^ oil. Sham animals received only sterile saline (days 0 and 7) and oil (days 11, 14–18, 21–24), while the naive group was only manipulated. After 24 h of the last OVA challenge, animals were anesthetized with ketamine hydrochloride (100 mg/kg, i.p.; Cetamin/Syntec) and xylazine hydrochloride (20 mg/kg; Xialazin/Syntec), then euthanized by cervical dislocation for skin collection.

### 2.2. Histological Analysis and Quantification of Mast Cells in Skin

Skins were fixed in 4% paraformaldehyde for 24 h and processed to paraffin embedding. Skin sections (3 µm) were stained by Diff-Quick (Laborclin, Brazil), a rapid hematology stain, which is also based on the Romanowsky technique. For quantification of mast cells, intact cells were characterized by metachromatic cytoplasmic granules, while degranulated cells by the exocytosis of granules in the dermis. Cells were quantified in 10 fields per section, using a 40× objective on an Axio Scope A1 Zeiss microscope (Carl Zeiss, Jena, Germany). Areas of each tissue were obtained using Axiovision software 4.8 (Carl Zeiss). Values are shown as mean ± standard error of the mean (SEM) of the number of cells per mm^2^.

### 2.3. Human Skin Biopsies

Paraffin-embedded human skin biopsies (*n* = 9), with confirmed AD clinical diagnosis and anatomopathological analysis, were provided by the Department of Pathology and Forensic Medicine, São José do Rio Preto School of Medicine (FAMERP), Brazil. For the control group, biopsies of clinically normal skin were used (*n* = 10). The study was approved by the Ethics Committee in Research of Faculdade de Medicina de São José do Rio Preto—FAMERP (CEP 2.225.518/2017), Brazil.

### 2.4. Galectin-9 Levels: Immunohistochemistry and Western Blotting

Immunohistochemistry and Western blot analysis were performed as described previously [13]. Briefly, for Gal-9 immunostaining, 3 µm-thick sections of mouse and human skins were incubated with polyclonal rabbit antibody anti-Gal-9 (Cusabio, College Park, MD, USA), diluted 1:4000 (mouse skin) or 1:200 (human skin) in PBS 1% bovine serum albumin (BSA) for 16–18 h, at 4 °C. After washing, sections were incubated with a secondary biotinylated antibody (LAB-SA Detection kit, Invitrogen, Paisley, UK). Positive staining was detected using a peroxidase-conjugated streptavidin complex, and color was developed using DAB substrate (Invitrogen). The sections were counterstained with hematoxylin. Densitometry analysis of Gal-9 immunostaining was performed in the epidermis and dermis (*n* = 5 animals/group or 9–10 patients/group). The values were obtained as arbitrary units (a.u.) between 0 and 255 using AxioVision software on an Axioskop 2 mot plus Zeiss microscope (Carl Zeiss, Jena, Germany). The data are expressed as the mean ± SEM of a.u.

For Western blotting, pooled protein extracts (30 µg per lane) of mouse skin (*n* = 3 animals per group) from indicated experimental conditions were loaded onto a 12% sodium dodecyl sulphate-polyacrylamide gel for electrophoresis together with appropriate molecular weight markers (Bio-Rad Life Science, Hercules, CA, USA) and transferred to ECL Hybond nitrocellulose membranes. Membranes were incubated for 15 min in 5% BSA in Tris-buffered saline (TBS) prior to incubation with rabbit polyclonal anti-Gal-9 (1:200) and anti-β-actin (1:5000) (Sigma-Aldrich, St. Louis, MO, USA), all diluted in TBS with 0.1% Tween 20. Following primary antibody incubation, membranes were washed with TBS and incubated with peroxidase-conjugated goat antirabbit IgG (1:2000) (Thermo Fisher Scientific Inc., Waltham, MA, USA). Immunoreactive proteins were detected (Westar Nova 2.0 chemiluminescent substrate kit; Cyanagen, Bologna, Italy) using a GeneGnome5 chemiluminescence detection system (SynGene, Cambridge, UK).

### 2.5. Immunofluorescence

Colocalization of Gal-9 and specific markers for mast cells and eosinophils in mouse skin was performed through incubation of sections with polyclonal antibody goat anti-mMCP6 (mouse mast cell protease 6; R&D Systems, Minneapolis, MN, EUA) or anti-EPX (eosinophil peroxidase; Santa Cruz Biotechnology, Dallas, TX, USA), diluted 1:300 in PBS 1% BSA for 16–18 h at 4 °C. Sections were washed in PBS and incubated for 1 h at room temperature with rabbit antigoat Ab conjugated with phycoerythrin (PE), 1:200 (Merck Millipore, Burlington, MA, USA). After washing in PBS, sections were incubated in PBS 4% BSA, 3% glycine for 1 h and, with rabbit anti-Gal-9 Ab (1:1000; Cusabio) for 4 h, at room temperature. Sections were washed in PBS, incubated with goat antirabbit Ab conjugated with fluorescein isothiocyanate (FITC), 1:300 (Merck Millipore). Sections were mounted with Fluoroshield™ containing DAPI (Sigma-Aldrich) and analyzed on a Nikon Eclipse Ci-S fluorescence microscope (Tokyo, Japan).

### 2.6. Human Keratinocyte Culture and Treatments

Human normal immortalized keratinocytes (HaCaT, CLS—Cell Line Service 300493), were grown in modified Dulbecco Eagle medium (DMEM) high glucose (4.5 g/L) (Sigma-Aldrich, St. Louis, MO, USA) with 4 mM l-glutamine and supplemented with 10% fetal bovine serum (FBS) (Cultilab, Br), 1 mM sodium pyruvate (Gibco), 0.1 mg/mL streptomycin, and 100 U/mL penicillin (Invitrogen, Paisley, UK). Upon reaching 80% confluence, cells were trypsinized, centrifuged, and resuspended with 2 mL of medium. Cells were counted and viability was tested using Trypan blue, before plating for different tests. 1 **×** 10^4^ cells/well were cultured in 96-well plates with 200 µL of DMEM + 10% FBS and incubated at 37 °C under a humid atmosphere with 5% CO_2_. After reaching 80% confluence (~48 h), cells were submitted to the following experimental conditions: control (growth media) or stimulated with TNF-α/IFN-γ (10 ng/mL), IL-4, or IL-17 (100 ng/mL), according to previous studies [12,13,14]. After 15 min, part of the cytokine-stimulated cells received human recombinant Gal-9 (Cusabio, College Park, MD, USA) at 100 or 500 ng/mL. Recombinant human TNF-α, IFN-γ, IL-4, and IL-17 were purchased from Peprotech (Rocky Hill, NJ, USA).

### 2.7. Proinflammatory Cytokine and RANTES/CCL5 Levels

Keratinocytes were cultured in a 96-well plate at concentration of 1 × 10^4^ cells/well. After 24 h under different experimental conditions, IL-6, IL-8, and RANTES (regulated upon activation, normal T cell expressed and secreted; also known as CCL5) levels were detected in the cell supernatants using commercially available ELISA kits (BD Biosciences, San Diego, CA, USA for IL-6 and IL-8; R&D Systems, Minneapolis, MN, USA for RANTES) according to the manufacturer’s instructions. Values are shown as mean ± SEM of the protein (pg/mL).

### 2.8. Cell Proliferation Assay

The proliferation of keratinocytes was detected by the 3-(4,5-dimethylthiazol-2-yl)-2,5-diphenyl tetrazolium bromide (MTT) assay. Cells were cultured in 96-well plates at a concentration of 1 × 10^4^ cells/well. After 24, 48, and 72 h, the supernatant was collected to add the culture medium with 10% MTT (10 µL/well) to each well, and as negative control, 10% MTT solution was added to wells without cells. The samples were incubated for 4 h at 37 °C. For solubilization of formazan crystals, 50 μL of dimethyl sulfoxide (DMSO) (Sigma-Aldrich) were added to each well and incubated for 10 min at 37 °C. The cell proliferation rate was calculated from optical density (OD540) values measured using the microplate reader ELISA EXL800 spectrophotometer (BioTek Instruments, Seoul, South Korea). The data was presented as a percentage of the control (*n* = 3/group in 2 independent experiments).

### 2.9. Scratch-Wound Assay

3 × 10^4^ cells were seeded in a 24-well plate and grown to subconfluency in growth media. A p200 pipette tip was used to scrape the cell monolayer in a straight line. Cells were washed twice with PBS to remove the debris. Immediately, cells were treated either with growth media (control), TNF-α/IFN-γ (10 ng/mL), IL-4, or IL-17 (100 ng/mL), with or without treatment with Gal-9 (100 or 500 ng/mL). Wound assays were observed after 6 to 48 h. The percentage decrease in the wound gaps was calculated using the Axiovision software (ZEISS) and normalized to the time 0 h of wounds. To calculate the difference of initial wound gaps, the wound closure of control group was set as 100% and the wound closures of other groups were calculated as relative percentages compared to the control group.

### 2.10. Bioinformatic Analysis

Four studies containing publicly available transcriptome data were selected from the Gene Expression Omnibus repository (GEOR): GSE120721 (https://www.ncbi.nlm.nih.gov/geo/query/acc.cgi, accessed on 11 March 2021)—skins from healthy (control), lesion, and nonlesion AD patients; GSE27533 (https://www.ncbi.nlm.nih.gov/geo/query/acc.cgi, accessed on 11 March 2021) —control and IL-17A-stimulated HaCaT cells for 12 h; GSE36287 (https://www.ncbi.nlm.nih.gov/geo/query/acc.cgi, accessed on 11 March 2021)—primary keratinocytes from three donors (subjects 1, 2, and 3) were either untreated (control) or exposed to cytokines (IL-4, IL-13, IL-17A, IFN-alpha, IFN-gamma, and TNF); GSE130588 (https://www.ncbi.nlm.nih.gov/geo/query/acc.cgi, accessed on 7 April 2021)—control (healthy patients), lesion, and nonlesion skins from AD patients treated weekly with subcutaneous doses of 200 mg of dupilumab for 16 weeks. Datasets were individually analyzed using the license-free algorithms implemented in the GEO2R tool (available at http://www.ncbi.nlm.nih.gov/geo/geo2r/, accessed on 1 January 2021) that allows users to compare different groups of samples in a GEO series to examine differentially expressed genes according to experimental conditions. GEO2R was applied to detect the Gal-9 gene (*LGALS9*) between different experimental conditions. The *p* values of gene expression after Log2 transformation were used to calculate the Z-score (individual value—population average/population standard deviation).

### 2.11. Statistical Analysis

The data were analyzed using GraphPad software version 9.00. The Kolmogorov–Smirnov test was used to determine the normality of the data. The data of the experimental groups were compared by means of analysis of variance (ANOVA One-way), followed by the application of the Kruskal–Wallis test for nonparametric samples or the Bonferroni test for parametric ones. For transcriptome data, student t-test was applied when comparing two groups. *p* values less than 0.05 were considered statistically significant.

## 3. Results

### 3.1. Gal-9 Levels Are Upregulated in Murine and Human AD

**Figure 2:**
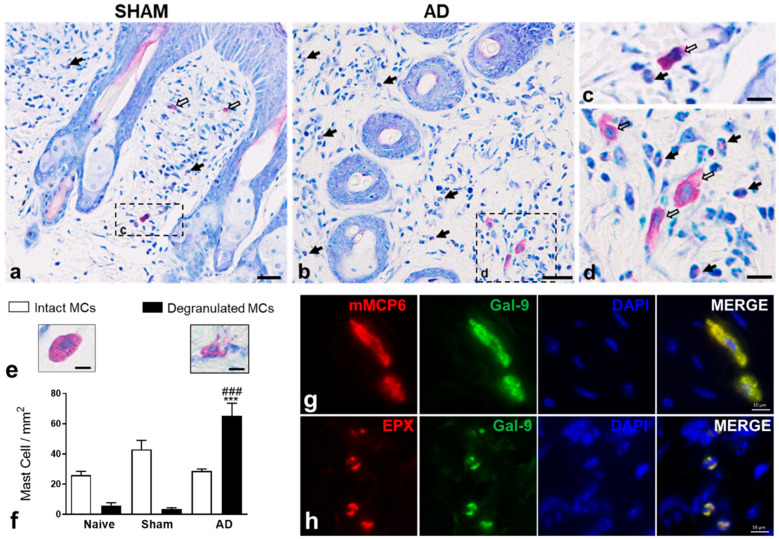
Inflammatory response in the skin. Intense influx of eosinophils (**b**,**d**; black arrows) and activated mast cells (**d**; white arrows) were observed in the (**b**) dermis of AD group in comparison to the (**a**) SHAM. Mast cells in (**d**) showed a weaker staining compared to the cell in (**c**) related to their greater activation with the release of cytoplasmic granules. Stain: Diff-Quick. (**e**,**f**) Quantification of intact (IMCs) and degranulated mast cells (DMCs) in the dermis. IMCs were characterized by metachromatic cytoplasmic granules, while DMCs by the exocytosis of granules in the dermis. Data represent mean ± SEM of the number of cells per mm^2^ (*n* = 5 animals/group). *** *p* < 0.001 vs. Naïve/Degranulated MCs; ^###^ *p* < 0.001 vs. Sham/Degranulated MCs (ANOVA, Bonferroni post-test). (**g**,**h**) Immunofluorescence double staining for mouse mast cell protease 6 (mMCP6) or eosinophil peroxidase (EPX) and Gal-9 in AD skin. mMCP6 and EPX are colocalized with Gal-9 in the cytosol of mast cells (**g**) and eosinophils (**h**). DAPI was used as a nuclear counterstain. Scale bars: 25 µm (**a,b**); 10 µm (**c,d,g,h**); 5 µm (**e**).

As previously described by Corrêa et al. [13], 24 h after the last OVA challenge, the skins of the mice showed epidermal hyperplasia patches and an intense influx of inflammatory cells in the dermis, especially eosinophils and mast cells, when compared to the control groups (naive and sham) (Figure 2a,b). In the AD group, several mast cells show a weaker staining, suggesting greater cell activation with the consequent release of mediators from cytoplasmic granules compared to the control condition (Figure 2c,d). The quantification of intact and degranulated mast cells in the dermis confirmed the histological observations. AD skin samples showed a significant increase in degranulated mast cells compared to controls, while the number of intact cells was similar between the different experimental groups (Figure 2e,f). AD skin samples were stained for the specific mast cell and eosinophil mediator markers, mMCP6 and EPX, respectively. Results showed that these two cell types represent potential sources of Gal-9, as demonstrated by the colocalization of these markers in the cytoplasm by immunofluorescence (Figure 2g,h).

**Figure 3:**
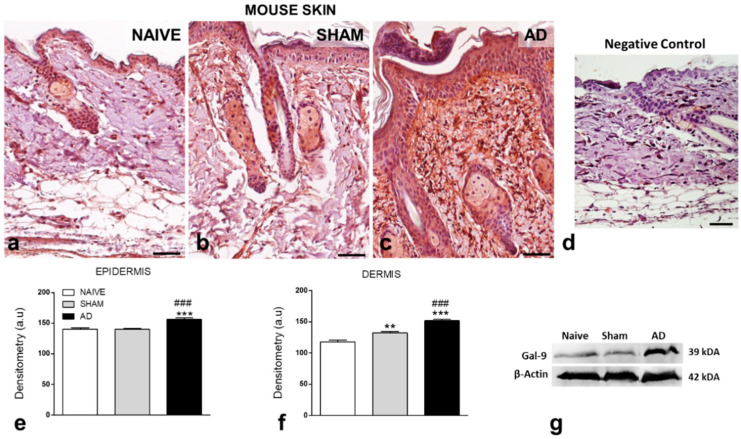
Expression of Gal-9 in the mouse skin. (**a**–**c**) Intense immunoreactivity for Gal-9 in the mouse epidermis and dermis detected in the AD group compared to controls (Naïve and Sham groups). (**d**) Negative control shows absence of immunoreactivity for Gal-9. Counterstain: hematoxylin. Scale bars: 40 µm. (**e**,**f**) Densitometric analysis of Gal-9 expression in mouse epidermis and dermis. Data represent means ± SEM of Gal-9 expression in arbitrary units (a.u.) (*n* = 5 animals/group). ** *p* < 0.01, *** *p* < 0.001 vs. Naive; ^###^ *p* < 0.001 vs. Sham (Kruskal–Wallis, Dunn post-test). (**g**) Western blot analysis to measure Gal-9 levels in the mouse skins. β-actin was used as a protein loading control (data represent one illustrative blot from two independent experiments).

In addition, Gal-9 expression was detected in the epidermis and dermis in all experimental conditions, showing the epithelium as a potential source of this lectin (Figure 3a–c). AD was associated with intense immunoreactivity for Gal-9 in the epidermis and dermis compared to the Naive and SHAM control groups (Figure 3a–c). No immunostaining was detected in the samples used to control the reaction (Figure 3d). These observations were confirmed by densitometric analysis, showing a significant increase in the levels of Gal-9 in the epidermis and dermis of the AD group compared to the controls (Figure 3e,f). Results from skin immunoblots corroborated these findings, showing strong immunoreactivity for Gal-9 levels in AD skin (Figure 3g).

**Figure 4:**
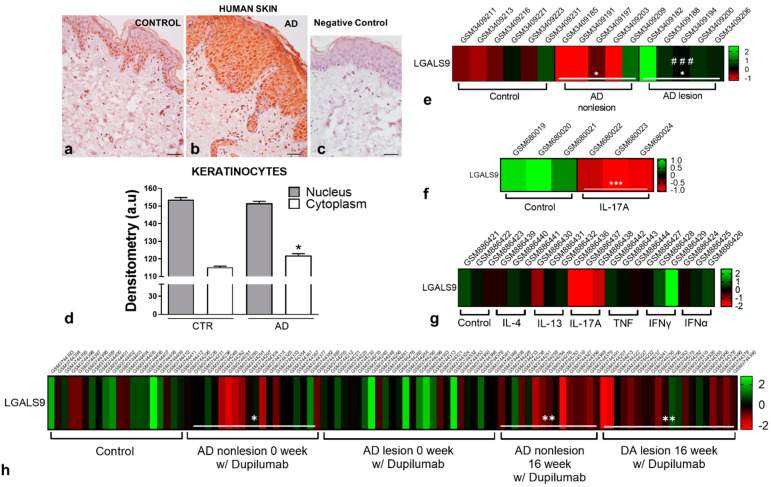
Expression of Gal-9 in the human skin. (**a**,**b**) Levels of Gal-9 in the human AD and control skins. Epidermis from AD skin samples shows intense immunoreactivity for Gal-9 compared to the control skin. (**c**) Absence of immunoreactivity for Gal-9 in the negative control. Counterstain: hematoxilin. Scale bars: 40 µm. (**d**) Densitometry for Gal-9 in keratinocytes. Data represent means ± SEM of Gal-9 expression in the nucleus and cytoplasm of cells in a.u. (*n* = 9–10 patients/group). * *p* < 0.05 vs. cytoplasm of control keratinocyte (Kruskal–Wallis, Dunn post-test). (**e**–**h**) Heatmaps based on the Z-scores of *LGALS9* transcriptional levels in the healthy (control) and AD human skins (GSE120721) (e); control/IL-17A-stimulated HaCaT cells (GSE27533) (**f**); control and cytokine (IL-4, IL-13, IL-17A, IFN-γ, IFN-α, and TNF)-stimulated primary keratinocytes (GSE36287) (**g**); and in the control (healthy patients), lesion, and nonlesion skins from AD patients treated weekly with subcutaneous doses of 200 mg of dupilumab for 16 weeks (GSE130588) (**h**). * *p* < 0.05, ** *p* < 0.01 vs. control (healthy skins); ^###^ *p* < 0.001 vs. AD nonlesion (ANOVA, Bonferroni post-test). *** *p* < 0.001 vs. control HaCaT cells (*t*-test, unpaired).

Expression of Gal-9 in human skin biopsies were analyzed in both control and AD patient samples. The analyses showed an increase in the expression of Gal-9 in the epidermis of AD skins compared to controls (Figure 4a,b). Negative control did not show immunopositivity for Gal-9, confirming the specificity of the primary antibody (Figure 4c). Densitometric scores confirmed the histological observations, showing an increase in the expression of Gal-9 in AD samples, especially in the cytoplasm of the keratinocytes (Figure 4d). Transcriptome analyses from the GSE120721 study also show increased levels of Gal-9 mRNA in lesion AD skins compared with nonlesion AD and control (healthy) skins (Figure 4e). Additionally, transcriptome analyses of HaCaT cells demonstrated decreased transcriptional levels of the *LGALS9* gene under IL-17A stimulation compared to the control cells (Figure 4f). A similar pattern of *LGALS9* expression was detected in the primary keratinocytes under IL-17A stimulation, however, under other inflammatory stimuli, the expression pattern of *LGALS9* changes completely (Figure 4g).

Interestingly, mRNA transcriptomics from the GSE130588 study showed that treatment with dupilumab, an efficient biologic therapy for AD that inhibits signaling of both IL-4 and IL-13 [14,15], downregulated the transcriptional levels of the *LGALS9* gene in lesion skins from AD patients compared to control skins (Figure 4h). Nonlesion AD skins also showed decreased levels of *LGALS9* in weeks 0 and 16 of drug treatment compared to control skins (Figure 4h).

### 3.2. Effect of Exogenous Administration of Gal-9 on Keratinocytes: Cytokine Release, Proliferation and Migration Rates

**Figure 5:**
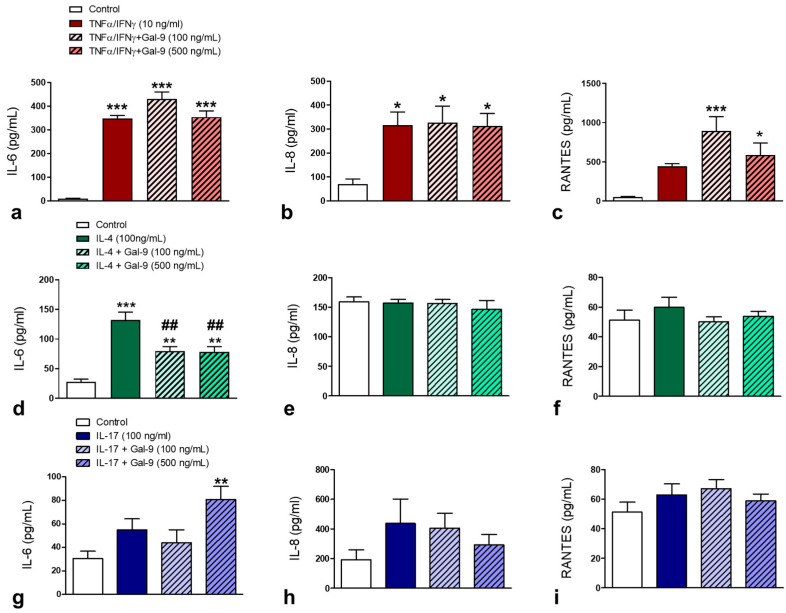
Effect of Gal-9 on cytokine release by keratinocytes. (**a**,**d**,**g**) IL-6 levels. (**b**,**e**,**h**) IL-8 levels. (**c**,**f**,**i**) RANTES levels (*n* = 3/group in 2 independent experiments). Human keratinocytes were submitted to the following experimental conditions: control (growth media) or stimulated with TNF-α/IFN-γ (10 ng/mL; (**a**)) IL-4 (100 ng/mL; (**d**)) or IL-17 (100 ng/mL; (**g**)), and after 15 min they received human recombinant Gal-9 at 100 or 500 ng/mL or media (control). After 24 h, ELISA was performed to determine cytokine release. Data are presented as the mean ± SEM of cytokine levels (pg/mL). * *p* < 0.05; ** *p* < 0.01; *** *p* < 0.001 vs. control; ^##^ *p* < 0.01 vs. at corresponding cytokine stimulation without Gal-9 (ANOVA, Bonferroni post-test).

Once it was found that the levels of Gal-9 mRNA and protein are increased in AD and after treatment with dupilumab they are reduced, our next step was to evaluate the effect of exogenous administration of Gal-9 on keratinocytes under different cytokine stimulation. After 24 h of TNF-α/IFN-γ stimulation, keratinocytes significantly increased IL-6 and IL-8 production compared to the control cells (Figure 5a,b), and no effect of Gal-9 treatment was observed for both cytokines under TNF-α/IFN-γ stimulation. Curiously, TNF-α/IFN-γ-stimulated keratinocytes plus Gal-9 treatment (100 and 500 ng/mL) produced a marked release of RANTES compared to control cells (Figure 5c). Under IL-4 stimulation, Gal-9 at 100 and 500 ng/mL significantly decreased the IL-6 release (Figure 5d). On the other hand, IL-17-stimulated keratinocytes plus Gal-9 treatment (500 ng/mL) produced a marked release of IL-6 compared to control cells (Figure 5g). No alterations were detected in the IL-8 and RANTES release by the IL-4- or IL-17-stimulated keratinocytes (Figure 5e,f,h,i).

**Figure 6:**
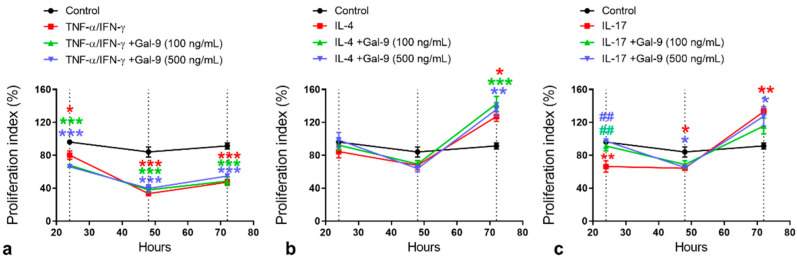
Effect of Gal-9 on HaCaT proliferation rate. Human keratinocytes were submitted to the following experimental conditions: control (growth media) or stimulated with TNF-α/IFN-γ (10 ng/mL; (**a**)) IL-4 (100 ng/mL; (**b**)) or IL-17 (100 ng/mL; (**c**)), and after 15 min they received human recombinant Gal-9 at 100 or 500 ng/mL or media. After 24, 48, and 72 h, an MTT assay was performed to determine proliferation rate (% of control; data represent mean ± SEM) (*n* = 3/group in 2 independent experiments). * *p* < 0.05; ** *p* < 0.01; *** *p* < 0.001 vs. control; ^##^ *p* < 0.01 vs. IL-17-stimulated cells (ANOVA, Bonferroni post-test).

The next step is to evaluate the effect of the Gal-9 on the keratinocyte proliferation rate. No effect of this lectin treatment was detected under TNF-α/IFN-γ and IL-4 stimulation. TNF-α/IFN-γ (with or without 100 or 500 ng/mL of Gal-9) produced a marked decrease of the keratinocyte proliferation rate, especially at 48 and 72 h, in comparison to control cells (Figure 6a). Under IL-4 stimulation (with or without Gal-9), an increased proliferation rate was detected for keratinocytes at 72 h (Figure 6b). On the other hand, both concentrations of Gal-9 increased keratinocyte proliferation at 24 h compared to the untreated IL-17-stimulated cells (Figure 6c). At 48 and 72 h, the lowest concentration of Gal-9 abrogated the effect of IL-17 stimulation on keratinocytes and produced similar proliferative rates of control cells (Figure 6c).

**Figure 7:**
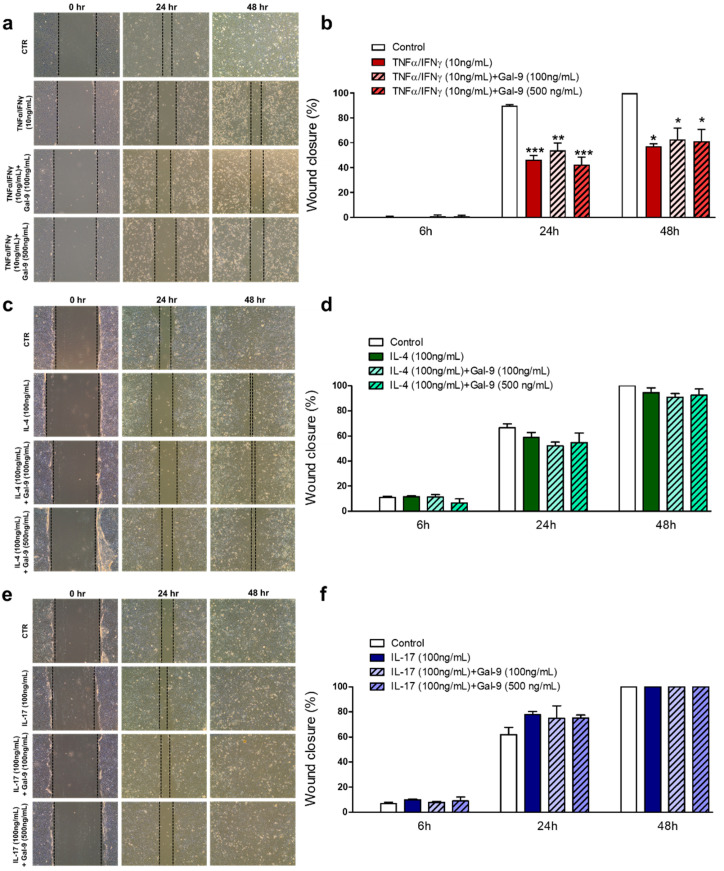
Scratch assay: assessment of HaCaT migration rate after cytokine stimulation and Gal-9 treatment. Human keratinocytes were submitted to the following experimental conditions: control (growth media), stimulated with TNF-α/IFN-γ (10 ng/mL), IL-4 (100 ng/mL), or IL-17 (100 ng/mL), and after 15 min they received human recombinant Gal-9 at 100 or 500 ng/mL or media. Wound assays were observed after 6, 24, and 48 h. Cells were seeded in complete media. Representative photomicrographs show no effect of Gal-9 treatment on keratinocytes under TNF-α/IFN-γ (**a**,**b**), IL-4 (**c**,**d**), or IL-17 (**e**,**f**) stimulation compared to nontreated conditions (*n* = 3/group in 2 independent experiments). Data are presented as the mean ± SEM of wound closure (%). * *p* < 0.05; ** *p* < 0.01; *** *p* < 0.001 vs. control at the corresponding time point (ANOVA, Bonferroni post-test).

We also verified the effect of Gal-9 on cell migration using the scratch-wound assay (Figure 7). TNF-α/IFN-γ stimulation, with or without Gal-9 treatment, significantly reduced the rate of keratinocyte migration compared to the control (Figure 7a,b), while no differences were detected under IL-4 and IL-17 stimulation alone or with Gal-9 treatment (Figure 7c–f).

## 4. Discussion

Considering that the role of Gal-9 is not well established in allergic inflammation, in this study, we evaluated the expression of this lectin in an experimental AD model in mice and human skin biopsies from patients with AD, as well as the effect of the administration of Gal-9 in human keratinocytes in vitro.

Histological analysis of the mice’ skin revealed intense eosinophilia in the dermis, in addition to a high influx of degranulated mast cells in AD animals when compared to controls (Naive and Sham). These findings corroborate other studies using an experimental model of AD induced by OVA [13,16], mite [17], dinitrochlorobenzene [18], and oxazolone [19], whose models mimic type I and IV hypersensitivity responses. In addition, studies using a model of pollen-induced allergic conjunctivitis have demonstrated that mast-cell-deficient mice, after been challenged with pollen in the conjunctival sac, had not shown clinical signs and conjunctival eosinophilia as prominently as in wild animals [20,21]. This effect was reversed when the deficient animals were repopulated with mast cells, showing that these cells have a prominent role in the initiation of the allergic response in conjunctivitis (clinical signs) and recruitment of eosinophils.

Inflammatory response observed in our murine model of AD was associated with high levels of Gal-9 when compared to the respective control groups. Interestingly, our results showed that mast cells and eosinophils are potential sources of Gal-9 in skin with AD. In fact, Gal-9 is expressed in human mast cell (HCM-1) and mouse (MC/9) cells lines that, upon stimulation, release this lectin to the external environment [9]. Peripheral blood eosinophils from patients with hypereosinophilic diseases (eosinophilic pneumonia, bronchial asthma, angiolymphoid hyperplasia with eosinophilia, and hypereosinophilic syndrome) have strong immunoreactivity for Gal-9 in the plasma membrane and cytoplasm compared to nonatopic control cells [22]. In experimental models of OVA- or mite-induced asthma in rodents, high levels of Gal-9 were detected in the lungs and bronchoalveolar fluid (BALF) 7 and 24 h after the last challenge [23,24,25]. An enhanced number of Gal-9-positive inflammatory cells in the BALF has also been detected in animals challenged with OVA [24].

In skin lesions of AD patients, we showed that Gal-9 is highly expressed in the epidermis compared with control skins, especially in the cytoplasm of keratinocytes, which is in line with previous studies [6,26]. The gene expression profiling analysis from the GSE120721 study showed increased levels of *LGALS9* in AD skin lesions compared with nonlesion AD and control (healthy) skins, which confirmed our immunohistochemistry findings. Furthermore, mRNA transcriptomics of Gal-9 from the GSE130588 study showed that treatment with dupilumab for 16 weeks downregulated its transcriptional levels in the lesional skins from AD patients compared to control skins. In this study, the inhibition of IL-4/IL-13 signaling through dupilumab treatment significantly improved AD signs and symptoms and induced a progressive shift of AD molecular phenotype from lesional to nonlesional skin [27]. Altogether, the studies confirm that the Th2-predominant inflammation induces a marked increase in the Gal-9 mRNA and protein levels in AD skins by keratinocytes (epidermis) and inflammatory cells (mast cells and eosinophils in the dermis), an effect abrogated by the inhibition of IL-4/IL-13 signaling.

Prevention of allergic symptoms was related with increased levels of Gal-9 in the intestinal epithelial cells induced by the ingestion of a dietary probiotic mixture or sym-biotic [28,29]. Using a murine model for cow’s milk allergy, the authors showed a reduction in acute hypersensitivity response associated with increased serum Gal-9 levels in allergic mice compared to the SHAM group [28]. Additionally, incubation of Gal-9 with human peripheral blood mononuclear cells from healthy donors enhanced the percentage of Th1 and Treg cells dose-dependently, resulting in increased secretion of IFN-γ and IL-10 and suppressed IL-17 production [28]. It has been shown Gal-9-treated monocyte-derived human dendritic cells also increased the secretion of Th1 cytokines (IFN, TNF, and IL-2) from allogeneic CD4 T cells, whereas no effect was detected in the production of Th2 (IL-4 and IL-5) [30], which can contribute to the suppression of allergen-induced Th2-type responses.

In fact, intravenous, intranasal, or sublingual administration of Gal-9 has been shown to be an important therapeutic tool in experimental models of asthma [25,31,32]. In these studies, treatment with Gal-9 reduced hyperactivity of respiratory epithelial cells, hypersecretion of mucus, leukocyte infiltration into the lungs, and BALF. It has also been shown that Gal-9 treatment reduced Th1 (TNF-α, IFN-γ) and Th2 (IL-5, IL-13) cytokine and chemokine (RANTES, IP-10, CCL11, and CCL17) production in BALF. On the other hand, intraperitoneal or subconjunctival treatments of mice with anti-Gal-9 antibodies do not affect the severity of pollen-induced allergic conjunctivitis, both in the induction and effector phases of the disease [33]. Overall, investigations demonstrate a potent anti-inflammatory role of Gal-9 in the responses induced by different allergens in the murine models of Th2-diseases and, when the protein is blocked, this effect is suppressed.

Regarding AD, epidermal barrier disruption and microbial invasion trigger the production of various keratinocyte-derived cytokines, such as IL-6, TNF-α, and RANTES, which promote the recruitment of inflammatory cells to the skin and activation of T helper (Th)17 cells, which further play an important part in the early stage of this disease [34]. Additionally, IL-17 stimulates Th2 cells to produce IL-4, contributing to the AD inflammation [35]. Curiously, IL-17 production can contribute to the downregulation of Gal-9 in the epidermis, as showed by the decreased levels of *LGALS9* in the IL-17A-stimulated human keratinocytes in the transcriptome analysis of GSE27533 and GSE36287 studies.

Considering that keratinocyte-derived cytokines are involved in the pathogenesis of AD [34,36], the next step was to evaluate the exogenous effect of Gal-9 in the human keratinocytes under a mimetic AD microenvironment (TNF-α/IFN-γ, IL-4, or IL-17 stimulation). After 24 h of inflammatory stimulation, keratinocytes showed an enhanced release of IL-6 and IL-8. The effect of Gal-9 on the release of cytokines by keratinocytes was antagonistic and dependent on the inflammatory stimulus involved. Under the effect of TNF-α/IFN-γ, exogenous administration of Gal-9 induced the release of RANTES by keratinocytes, which contributes to the recruitment of eosinophils in AD skin [37]. Treatment with Gal-9 was also able to reverse the release of IL-6 in the IL-4-stimulated cells, but under IL-17 stimulation, the higher concentration of Gal-9 produced greater release of IL-6 by keratinocytes. Considering that (i) Th2 cytokines can stimulate eosinophils to produce IL-12 and promote a switch from a Th2-like immune response in acute lesions to a Th1-like immune response in chronic lesions of AD [38,39], and (ii) IL-6 contributes to the transition from acute to chronic phase of AD through induction of Th2 differentiation and inhibition of Th1 polarization [33], our findings show a potent immunomodulatory effect of Gal-9 on keratinocytes under the inflammatory microenvironment of AD.

Finally, under IL-17 stimulation, we showed that exogenous Gal-9 at both concentrations (100 and 500 ng/mL) increased keratinocyte proliferation rate at 24 h, an effect that was abrogated in a dependent concentration manner. However, Gal-9 treatment showed no effect on the keratinocyte proliferation and migration rates under IL-4 and TNF-α/IFN-γ stimulation. Despite this, studies have shown that IFN-γ suppresses mRNA expression of Gal-9 in epidermal keratinocytes, as well as reduces surface Gal-9 expression in a dose-dependent manner [12]. Furthermore, transfection of Gal-9 cDNA into oral squamous carcinoma Ca9-22 cells produced a marked increase in the cellular adhesion to fibronectin and collagen I compared to nontransfected cells [40].

Altogether, these findings suggest that exogenous Gal-9 exerts an immunomodulatory effect on keratinocytes and contributes to epidermal homeostasis through regulation of cell adhesion and proliferation against increased epidermal proliferation and disturbed differentiation provoked by AD-induced inflammatory microenvironment. However, this study has some limitations and more detailed investigations are warranted in the future to evaluate the effect of Gal-9 administration in the IL-13 and IL-22-stimulated keratinocytes, two important cytokines that regulates cellular responses in AD pathogenesis [41].

## 5. Conclusions

In conclusion, our results showed that increased levels of Gal-9 in the pathogenesis of AD represent an important step for counter-regulation of skin inflammatory response and epidermis proliferation.

## Data Availability

Data available on request due to restrictions.

## References

[1] Leung D.Y., Guttman-Yassky E. (2014). Deciphering the complexities of atopic dermatitis: Shifting paradigms in treatment approaches. J. Allergy Clin. Immunol..

[2] Guttman-Yassky E., Krueger J.G. (2017). Atopic dermatitis and psoriasis: Two different immune diseases or one spectrum?. Curr. Opin. Immunol..

[3] Dainichi T., Kitoh A., Otsuka A., Nakajima S., Nomura T., Kaplan D.H., Kabashima K. (2018). The epithelial immune microenvironment (EIME) in atopic dermatitis and psoriasis. Nat. Immunol..

[4] Elola M.T., Ferragut F., Méndez-Huergo S.P., Croci D.O., Bracalente C., Rabinovich G.A. (2018). Galectins: Multitask signaling molecules linking fibroblast, endothelial and immune cell programs in the tumor microenvironment. Cell Immunol..

[5] Liu F.T., Rabinovich G.A. (2010). Galectins: Regulators of acute and chronic inflammation. Ann. N. Y. Acad. Sci..

[6] Nakajima R., Miyagaki T., Oka T., Nakao M., Kawaguchi M., Suga H., Morimura S., Kai H., Asano Y., Tada Y. (2015). Elevated serum galectin-9 levels in patients with atopic dermatitis. J. Dermatol..

[7] Niwa H., Satoh T., Matsushima Y., Hosoya K., Saeki K., Niki T., Hirashima M., Yokozeki H. (2009). Stable form of galectin-9, a Tim-3 ligand, inhibits contact hypersensitivity and psoriatic reactions: A potent therapeutic tool for Th1- and/or Th17-mediated skin inflammation. Clin. Immunol..

[8] Cedeno-Laurent F., Barthel S.R., Opperman M.J., Lee D.M., Clark R.A., Dimitroff C.J. (2010). Development of a nascent galectin-1 chimeric molecule for studying the role of leukocyte galectin-1 ligands and immune disease modulation. J. Immunol..

[9] Niki T., Tsutsui S., Hirose S., Aradono S., Sugimoto Y., Takeshita K., Nishi N., Hirashima M. (2009). Galectin-9 is a high affinity IgE-binding lectin with anti-allergic effect by blocking IgE-antigen complex formation. J. Biol. Chem..

[10] Kojima R., Ohno T., Iikura M., Niki T., Hirashima M., Iwaya K., Tsuda H., Nonoyama S., Matsuda A., Saito H. (2014). Galectin-9 enhances cytokine secretion, but suppresses survival and degranulation, in human mast cell line. PLoS ONE.

[11] Sato M., Nishi N., Shoji H., Seki M., Hashidate T., Hirabayashi J., Kasai Ki K., Hata Y., Suzuki S., Hirashima M. (2002). Functional analysis of the carbohydrate recognition domains and a linker peptide of galectin-9 as to eosinophil chemoattractant activity. Glycobiology.

[12] Igawa K., Satoh T., Hirashima M., Yokozeki H. (2006). Regulatory mechanisms of galectin-9 and eotaxin-3 synthesis in epidermal keratinocytes: Possible involvement of galectin-9 in dermal eosinophilia of Th1-polarized skin inflammation. Allergy.

[13] Corrêa M.P., Andrade F.E.C., Gimenes A.D., Gil C.D. (2017). Anti-inflammatory effect of galectin-1 in a murine model of atopic dermatitis. J. Mol. Med. Berl..

[14] Beck L.A., Thaçi D., Hamilton J.D., Graham N.M., Bieber T., Rocklin R., Ming J.E., Ren H., Kao R., Simpson E. (2014). Dupilumab treatment in adults with moderate-to-severe atopic dermatitis. N. Engl. J. Med..

[15] Zhou S., Qi F., Gong Y., Zhang J., Zhu B. (2021). Biological Therapies for Atopic Dermatitis: A Systematic Review. Dermatology.

[16] Kim H.J., Kim Y.J., Kang M.J., Seo J.H., Kim H.Y., Jeong S.K., Lee S.H., Kim J.M., Hong S.J. (2012). A novel mouse model of atopic dermatitis with epicutaneous allergen sensitization and the effect of Lactobacillus rhamnosus. Exp. Dermatol..

[17] Matsuoka H., Maki N., Yoshida S., Arai M., Wang J., Oikawa Y., Ikeda T., Hirota N., Nakagawa H., Ishii A. (2003). A mouse model of the atopic eczema/dermatitis syndrome by repeated application of a crude extract of house-dust mite Dermatophagoides farinae. Allergy.

[18] Kim H., Kim J.R., Kang H., Choi J., Yang H., Lee P., Kim J., Lee K.W. (2014). 7,8,4’-Trihydroxyisoflavone attenuates DNCB-induced atopic dermatitis-like symptoms in NC/Nga mice. PLoS ONE.

[19] Heo W.I., Lee K.E., Hong J.Y., Kim M.N., Oh M.S., Kim Y.S., Kim K.W., Kim K.E., Sohn M.H. (2015). The role of interleukin-17 in mouse models of atopic dermatitis and contact dermatitis. Clin. Exp. Dermatol..

[20] Miyazaki D., Tominaga T., Yakura K., Kuo C.H., Komatsu N., Inoue Y., Ono S.J. (2008). Conjunctival mast cell as a mediator of eosinophilic response in ocular allergy. Mol. Vis..

[21] Fukuda K., Ohbayashi M., Morohoshi K., Zhang L., Liu F.T., Ono S.J. (2009). Critical role of IgE-dependent mast cell activation in a murine model of allergic conjunctivitis. J. Allergy Clin. Immunol..

[22] Saita N., Goto E., Yamamoto T., Cho I., Tsumori K., Kohrogi H., Maruo K., Ono T., Takeya M., Kashio Y. (2002). Association of galectin-9 with eosinophil apoptosis. Int. Arch. Allergy Immunol..

[23] Yamamoto H., Kashio Y., Shoji H., Shinonaga R., Yoshimura T., Nishi N., Nabe T., Nakamura T., Kohno S., Hirashima M. (2007). Involvement of galectin-9 in guinea pig allergic airway inflammation. Int. Arch. Allergy Immunol..

[24] Sziksz E., Kozma G.T., Pállinger E., Komlósi Z.I., Adori C., Kovács L., Szebeni B., Rusai K., Losonczy G., Szabó A. (2010). Galectin-9 in allergic airway inflammation and hyper-responsiveness in mice. Int. Arch. Allergy Immunol..

[25] Katoh S., Shimizu H., Obase Y., Oomizu S., Niki T., Ikeda M., Mouri K., Kobashi Y., Hirashima M., Oka M. (2013). Preventive effect of galectin-9 on double-stranded RNA-induced airway hyperresponsiveness in an exacerbation model of mite antigen-induced asthma in mice. Exp. Lung Res..

[26] Farag A.G.A., Al-Sharaky D.R., Allam S.S., Khaled H.N. (2020). Role of Galectin-9 in Atopic Dermatitis—Is It Mediated Through E Selectin? A Clinical and Immunohistochemical Study. Clin. Cosmet Investig. Dermatol..

[27] Guttman-Yassky E., Bissonnette R., Ungar B., Suárez-Fariñas M., Ardeleanu M., Esaki H., Suprun M., Estrada Y., Xu H., Peng X. (2019). Dupilumab progressively improves systemic and cutaneous abnormalities in patients with atopic dermatitis. J. Allergy Clin. Immunol..

[28] De Kivit S., Saeland E., Kraneveld A.D., van de Kant H.J., Schouten B., van Esch B.C., Knol J., Sprikkelman A.B., van der Aa L.B., Knippels L.M. (2012). Galectin-9 induced by dietary synbiotics is involved in suppression of allergic symptoms in mice and humans. Allergy.

[29] Kim H.W., Ju D.B., Kye Y.C., Ju Y.J., Kim C.G., Lee I.K., Park S.M., Choi I.S., Cho K.K., Lee S.H. (2019). Galectin-9 Induced by Dietary Probiotic Mixture Regulates Immune Balance to Reduce Atopic Dermatitis Symptoms in Mice. Front Immunol..

[30] Dai S.Y., Nakagawa R., Itoh A., Murakami H., Kashio Y., Abe H., Katoh S., Kontani K., Kihara M., Zhang S.L. (2005). Galectin-9 induces maturation of human monocyte-derived dendritic cells. J. Immunol..

[31] Ikeda M., Katoh S., Shimizu H., Hasegawa A., Ohashi-Doi K., Oka M. (2017). Beneficial effects of Galectin-9 on allergen-specific sublingual immunotherapy in a Dermatophagoides farinae-induced mouse model of chronic asthma. Allergol. Int..

[32] Katoh S., Ishii N., Nobumoto A., Takeshita K., Dai S.Y., Shinonaga R., Niki T., Nishi N., Tominaga A., Yamauchi A. (2007). Galectin-9 inhibits CD44-hyaluronan interaction and suppresses a murine model of allergic asthma. Am. J. Respir. Crit. Care Med..

[33] Diehl S., Rincón M. (2002). The two faces of IL-6 on Th1/Th2 differentiation. Mol. Immunol..

[34] Chieosilapatham P., Kiatsurayanon C., Umehara Y., Trujillo-Paez J.V., Peng G., Yue H., Nguyen L.T.H., Niyonsaba F. (2021). Keratinocytes: Innate immune cells in atopic dermatitis. Clin. Exp. Immunol..

[35] Nakajima S., Kitoh A., Egawa G., Natsuaki Y., Nakamizo S., Moniaga C.S., Otsuka A., Honda T., Hanakawa S., Amano W. (2014). IL-17A as an inducer for Th2 immune responses in murine atopic dermatitis models. J. Investig. Dermatol..

[36] Asahina R., Maeda S. (2017). A review of the roles of keratinocyte-derived cytokines and chemokines in the pathogenesis of atopic dermatitis in humans and dogs. Vet. Dermatol..

[37] Kameyoshi Y., Dörschner A., Mallet A.I., Christophers E., Schröder J.M. (1992). Cytokine RANTES released by thrombin-stimulated platelets is a potent attractant for human eosinophils. J. Exp. Med..

[38] Liu F.T., Goodarzi H., Chen H.Y. (2011). IgE, mast cells, and eosinophils in atopic dermatitis. Clin. Rev. Allergy Immunol..

[39] Grewe M., Czech W., Morita A., Werfel T., Klammer M., Kapp A., Ruzicka T., Schöpf E., Krutmann J. (1998). Human eosinophils produce biologically active IL-12: Implications for control of T cell responses. J. Immunol..

[40] Kasamatsu A., Uzawa K., Nakashima D., Koike H., Shiiba M., Bukawa H., Yokoe H., Tanzawa H. (2005). Galectin-9 as a regulator of cellular adhesion in human oral squamous cell carcinoma cell lines. Int. J. Mol. Med..

[41] Brunner P.M., Guttman-Yassky E., Leung D.Y. (2017). The immunology of atopic dermatitis and its reversibility with broad-spectrum and targeted therapies. J. Allergy Clin. Immunol..

